# Predictors of Change in Play-Based Communication and Behavior Intervention for High-Risk ASD: The Role of Mother-Child Dyadic Synchrony

**DOI:** 10.3389/fped.2020.581893

**Published:** 2020-12-02

**Authors:** Linyan Fu, Jiao Weng, Min Feng, Xiang Xiao, Ting Xiao, Junli Fu, Nana Qiu, Chunyan Li, Yun Da, Xiaoyan Ke

**Affiliations:** ^1^Child Mental Health Research Center, Nanjing Brain Hospital Affiliated to Nanjing Medical University, Nanjing, China; ^2^Three Hospital of Longyan, Longyan, China

**Keywords:** autism, intervention efficacy, predictors, mother-child dyadic synchrony, parenting stress, play-based communication and behavior intervention

## Abstract

**Background:** Interindividual variability is important in the evolution of adaptative profiles of children with ASD having benefited from an early intervention make up for deficits in communication, language and social interactions. Therefore, this paper aimed to determine the nature of factors influencing the efficacy variability of a particular intervention technique i.e., “Play-based communication and behavior intervention” (PCBI).

**Methods:** The participants comprised 70 13–30-month-old toddlers with ASD enrolled in PCBI for 12 weeks. The Autism Treatment Evaluation Checklist (ATEC) was used to evaluate the efficacy of PCBI. Video recordings of 5 min of free-play before and after PCBI were used to examine behaviors of mothers and children and parent-child dyadic synchrony. Hierarchical multiple regression analyses and machine learning algorithms were performed to explore the effect of these potential predictors (mothers' factors, children's factors and videotaped mother-child interaction) of intervention efficacy.

**Results:** The hierarchical regression analysis and the machine learning algorithms indicated that parenting stress, level of completion of training at home and mother-child dyadic synchrony were crucial factors in predicting and monitoring the efficacy of PCBI.

**Conclusions:** In summary, the findings suggest that PCBI could be particularly beneficial to children with ASD who show a good performance in the mother-child dyadic synchrony evaluation. A better dyadic mother-child synchrony could enhance the PCBI efficacy through adapted emotional and behavioral responses of the mother and the child and has a beneficial influence on the child's psychological development.

## Introduction

Autism spectrum disorder (ASD) is a neurodevelopmental disorder characterized by difficulties in social communication and restricted/repetitive behavior or interests (1). Evidence of brain development and key stages of neural plasticity in infancy lends strong support to the importance of very early interventions (2). The first 2 years after birth is a critical period marked by the rapid development of cognition and language (3, 4). Children later diagnosed with ASD may present some deficits in these abilities (5), and the gap between these children and typically developing peers widens at 3–5 years of age (6). Studies have shown that early intervention is efficacious in modifying cognitive and social-communicative outcomes and potentially improving developmental trajectories in toddlers with ASD (4, 7). However, the majority of established interventions have been suggested for use with preschoolers and school-aged children, with very few interventions suitable for children under 2 years old (5).

The play-based communication and behavior intervention model (PCBI) is a manualized evidence-based intervention established by the Nanjing Brain Hospital affiliated with Nanjing Medical University. PCBI is a parent education play-based applied behavior analysis (ABA) model that fosters parental behavior management and parents' communication ability with their children. The characteristic of PCBI is to train parents or other caregivers to effectively master the key intervention techniques applicable to children with ASD, while the feature of ABA is to train ASD children directly. Our team's previous work confirmed that PCBI can significantly improve the development level of children with ASD and reduce ASD symptoms [effect size = 0.88, (8)]. In our previous study, 74 toddlers were randomly assigned to receive PCBI or ABA intervention from highly experienced and credentialed therapists. After 12 weeks of intervention, both groups had a significant increase in the developmental quotient (language behavior ability and personal-social behavior ability) and a decrease in the subscale scores (social contact, perception and behavior) of the Autism Treatment Evaluation Checklist (ATEC) (8).

Empirical support for early intervention has emphasized the importance of this treatment. However, outcomes vary widely due to the influence of child and family characteristics. For example, there is evidence that parents of children with ASD may experience high levels of parenting stress (9, 10), which can have a negative influence on the effectiveness of early interventions and can also be influenced by early interventions (11–13). In addition, parent-child dyadic synchrony, which refers to the co-constructed states of parent-child-dyad interaction characterized by harmonious and mutually responsive emotional exchanges and behavior, is a key component of parent-child interaction and can have positive implications for child development (14, 15). The conceptualizations of parent-child dyadic synchrony encompass an array of interactive behaviors, such as responsiveness, reciprocity and shared emotion evaluated during face-to-face interactions (16). The development of children's emotional regulation occurs through repeated emotional experiences with parents, and shared emotion, as an aspect of dyadic synchrony that lays the foundation for the development of subsequent self-regulation, may differ between children with ASD and typically developing children (17–19). Although parent-child dyadic synchrony plays an important role in children's development, studies have only recently focused on it and regarded it as an important indicator of parent-child relationship quality (20), and few researchers have suggested that parent-child synchrony should be considered a factor in the efficacy of interventions for children with ASD.

Because early intervention requires considerable time and funding, it is crucial to select the target group that is most likely to derive maximal benefit from the intervention. Thus, the primary purpose of this study was to extend our previous work to investigate all the potential predictors of the efficacy of PCBI, including maternal variables (parenting stress and self-efficacy level), child variables (developmental level, ASD symptoms and intervention efficacy change) and mother-child interaction factors (mother and child behaviors during interaction).

## Methods

### Participants

A total of 70 children (55 boys and 15 girls) ranging in age between 13 and 30 months and their mothers (between the ages of 25 and 42 years) who received PCBI at the Children's Mental Health Research Center of the Nanjing Brain Hospital affiliated with Nanjing Medical University from October 2017 to February 2019 were enrolled in this study. Because this is a preliminary study aiming to examine the clinical reliability/ validity of the protocol, all the participants received PCBI without a control group.

The participants were 70 children with ASD or at high risk of ASD (i.e., who displayed symptoms of autism but were under 24 months of age) between the ages of 8 and 30 months old at intake. Inclusion criteria included: met criteria for ASD in a clinical assessment based on both the Autism Diagnostic Observational Schedule (ADOS) and the Autism Diagnostic Interview (ADI-R) at intake, or met risk criteria for ASD at intake (under 24 months of age) on two screeners and met criteria for ASD later at the age of 24 months. Participants with any genetic syndromes or neurological conditions; premature children and those with complications after birth; a history of craniocerebral trauma; chronic medical conditions; or visual, hearing or motor impairments were not included in this study. This study was approved by the Nanjing Brain Hospital affiliated with the Nanjing Medical University Ethics Committee, and informed consent was obtained from the parents or legal guardians of the participants.

### Screening Tools and ASD Diagnosis

All the children were screened at enrollment and at the end of intervention by the M-CHAT and the Autism Behavior Checklist [ABC, (21)]. The M-CHAT is a 23-item parent questionnaire developed to screen children ranging in age from 16 to 30 months old (22). The reliability of M-CHAT is 94% in the Chinese population (23). The ABC is a well-established parent report checklist used to screen and diagnose autism. The higher the subscale and total ABC score are, the more significant the ASD symptoms are. Participant who did not reach the evaluation age of the screening tools when entering the group will be evaluated at the end of the intervention. The reliability and validity of ABC scale in the Chinese population are nearly 100% (24).

The ADOS is a semistructured, standardized diagnostic observational instrument that provides the diagnosis of ASD based on the DSM-IV algorithm. The ADOS is designed to assess autism symptoms in communicative behaviors, social reciprocity, repetitive behaviors and play (25). All the children received ADOS module 1 and ADI-R (26) to inform the ASD diagnosis, and the assessment was conducted at age 2 for participants under 2 years of age at the end of the intervention.

### Intervention

The caregivers received two and a half days of parent training before participating in PCBI to gain some understanding of the importance of early intervention and parental involvement. The PCBI consisted of 12 parent-child sessions, with one 1-h session per week. The staff-child ratio was 1:1. In sessions 1-11, the caregivers were coached in each of eleven intervention techniques from PCBI, addressing one new skill each week and improving those taught previously. The 11 skills were as follows: (a) behavioral training, focusing on positive behavior; (b) behavioral training, dealing with problem behavior; (c) behavioral training, developing training plans; (d) behavioral training, executing training plans; (e) communication training, facilitating joint attention; (f) communication training, responding to joint attention; (g) communication training, promoting speech development; (h) communication training, creating a language environment; (i) communication training, teaching simple game skills; (j) communication training, teaching functional play skills; and (k) communication training, teaching pretend play skills. Session 12 was devoted to maintenance after treatment and discussion of the follow-up training plan. The 60-min period of a session was roughly divided into three sections, each of which was 20 min long. In the first 20 min, the mother demonstrated the skills from the last lesson and the training at home, and the therapist provided feedback and suggestions. In the second 20 min, the therapist introduced some theoretical knowledge to the mother and used a demo game to interact with the child. During the last 20 min, the mother interacted with the child based on the interactive skills just observed, and the therapist provided timely guidance and feedback.

To ensure the effectiveness of the training, mothers were required to complete a feedback form about the lesson, continue training at home every day and submit a 10-min video recording about the training every week. The therapist rated the video clips and provided timely guidance and feedback to the parents. Only the mother with a score over 80 were considered to have mastered the lesson.

### Child Measures

#### Developmental Measures

The Gesell Developmental Schedules (27) is a standardized early developmental quotient (DQ) assessment that provides an overall index of ability on the following five subscales: adaptive behavior, gross motor behavior, language behavior, fine motor behavior and personal-social behavior. All the children were administered the Gesell at baseline and after intervention.

#### Efficacy Assessment

The Autism Treatment Evaluation Checklist [ATEC, (28)] was used to measure the efficacy of PCBI. It has been successfully used to assess the effectiveness of treatments and progress after intervention in ASD (29–31). The ATEC is designed to be self-administered by parents and elicits parent concerns in four domains: speech/language/communication (14 items, scores range from 0 to 28), health/physical behavior (25 items, scores range from 0 to 75), sensory/cognitive awareness (18 items, scores range from 0 to 36), and sociability (20 items, scores range from 0 to 40) (32). The total scores of these four subscales range from 0 to 180, and a higher subscale and total score indicate greater impairment in children. The reliability of ATEC is 75% and the validity is 92% in the Chinese population (33).

### Parent Measures

**The Parenting Stress Index Short Form (PSI-SF)** is a Likert-based 5-point self-report questionnaire with 36 items used to evaluate parenting stress (34). The PSI-SF consists of three subscales, parenting distress (PD), difficult child (DC) and parent-child dysfunctional interaction (PCDI), as well as a total stress scale. It has been used in previous studies with children with ASD and shows good reliability and validity (35, 36).

**The General Self-Efficacy Scale (GSES)** is widely implemented worldwide and was developed to measure an individual's self-efficacy level (37). Self-efficacy is defined as an individual's belief about his or her ability to control events related to the individual to meet a specified level of performance. A Chinese version (38) that consisted of 10 questions was used in this study.

### Acceptability and Fidelity

Mother-perceived acceptability is especially important for interventions when the mother is the primary interventionist. Satisfaction questionnaires were used to assess parental satisfaction with the intervention after each session. The therapist monitored treatment fidelity by rating mother's performance in correctly implementing techniques and adhering to intervention procedures during each session via a Likert-based 10-point rating system. We also evaluated the completion level of training at home via videotaped observation ratings.

### Mother-Child Interaction

**Figure 1:**
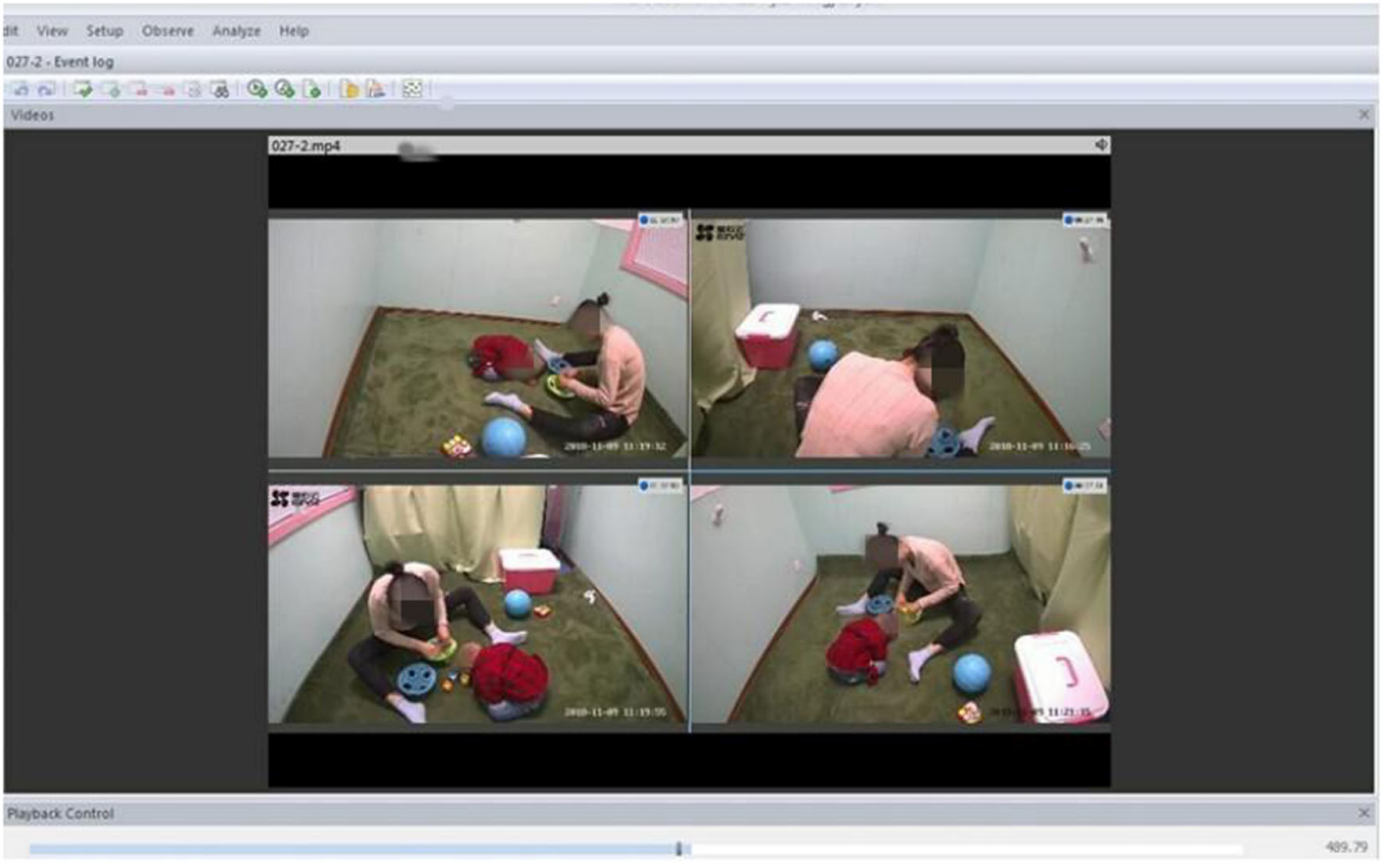
This figure provides an illustration of free play.

Mother-child interaction was assessed and videotaped with the mother and child sitting on a floor mat in a laboratory room (3 × 3 m^2^) at baseline and after intervention. The free play-based setting included a standard set of toys, such as cause-and-effect toys, books, dolls, balls, toy cars, toys for imaginative play (e.g., blocks), and a small towel for peekaboo games. At the beginning of the experiment, the child was given 3 min to independently explore three toys selected by the mother. Then, the mother was asked to engage in play with all the toys if the child wished as they normally would for the next 10 min (see Figure 1). Mother-child interaction was video recorded for later coding by a desktop computer, and four cameras were fixed to the wall. Because the toddlers varied widely in the way they explored the toys independently and the interaction in the last 5 min was relatively more stable, coding focused on the last 5 min of toddlers' free play with their mothers.

### Interaction Coding

Videotapes of the last 5 min of the mother-child interaction were rated on a 5-s interval basis using the Observer XT 12.0 behavioral coding software. The coding was performed by two independent coders. Interrater reliability was tested by coding 20% of the video recordings independently using Cohen's kappa interrater agreement coefficient. Cohen's kappa averaged 0.80 for mother and child behaviors. The coding schemes were designed with reference to the work of Guo et al. (18).

The coders rated mother and child behavior using 1- to 3-point rating scales, including positive engagement, negative engagement, disengagement, child-object engagement, and mother-child dyadic synchrony. *Mother positive engagement* refers to mothers expressing positive or neutral affect, vocalizations and behavior to interact with toddlers, such as playing an active imaginative game with a full smile on the face. *Mother negative engagement* refers to mothers expressing angry, hostile, irritable, or negative vocalizations to children as well as intruding on children's exploration. *Mother disengagement* refers to mothers expressing boredom or disinterest and avoiding interaction with their child. *Child positive engagement* refers to toddlers maintaining free play with their mothers by showing positive or neutral affect, vocalizations and positive body posture. *Child negative engagement* refers to toddlers expressing impatience, anger, distressed vocalizations and negative body posture to protest against their mothers, such as crying and pushing their mothers away. *Child disengagement* refers to toddlers withdrawing from a joint activity by expressing sadness or anxiety. *Child-object engagement* refers to toddlers focusing on exploring toys independently and without interacting with their mothers. *Mother-child dyadic synchrony* means that both mothers and children are in a positive state of engagement at the same time.

### Statistical Analysis

Data analyses were performed using SPSS 23.0. We evaluated changes in the mothers' variables, including the PSI-SF and GSES scores as well as the acceptability and fidelity of PCBI between baseline (T1) and postintervention (T2). The mother-child interaction variables and children's variables such as developmental level, ASD symptoms and intervention efficacy change were also assessed. Paired *t*-tests was used for data that obey normal distribution and non-parametric test was used for variables did not obey normal distribution. Next, hierarchical multiple regression analyses were performed to explore the effect of these potential predictors of intervention efficacy. All the variables were divided into two categories (children's and mothers' factors, mother-child interaction factors) to examine the main predictors in each category.

We also used machine learning algorithms to verify the factors that can predict the efficacy of intervention. LASSO regression, support vector machine (SVM), ridge regression (RR) and random forest (RF) algorithms were applied to develop regression modes in this study. Machine learning techniques were completed using Python (Python 3.6.5) and MATLAB's Statistics (MATLAB R2018a). We considered all the variables of mothers and children and the mother-child interaction as features, including the age of the mother and child; the subscale and total score on the ABC, Gesell, PSI-SF, and GSES; and both mothers' and children's behaviors during the videotaped mother-child interactions. A 5-fold cross-validation method was used for internal validation to ensure an unbiased estimate of the classifier performance. It consisted of dividing the entire group of 70 children randomly into 5 subsets of equal size; then, 4-folds were used as the training dataset and the remaining fold as the testing dataset. Then, all the features were sorted according to the weight, and the top five features were listed for each regression model. Root mean square error (RMSE) and *R*^2^ were used to describe and estimate the performance of the regression models. RMSE represents the differences between predicted and observed values, and *R*^2^ measures how well the model fits the predicted values; the better the model is, the closer the *R*^2^ is to 1. RMSE and *R*^2^ are defined via the following formulas:

$$\begin{array}{l} RMSE=\sqrt{\frac{\sum_{i=1}^{N}{\left(Y_{i}-X_{i}\right)}^{2}}{N}},\\ R^{2}=1-\frac{SS_{\text{res}}}{SS_{tot}}=1-\frac{\sum_{i}{\left(X_{i}-Y\right)}^{2}}{\sum_{i}{\left(X_{i}-\bar{X}\right)}^{2}} \end{array}$$

## Results

### Child Outcomes

**Figure 2:**
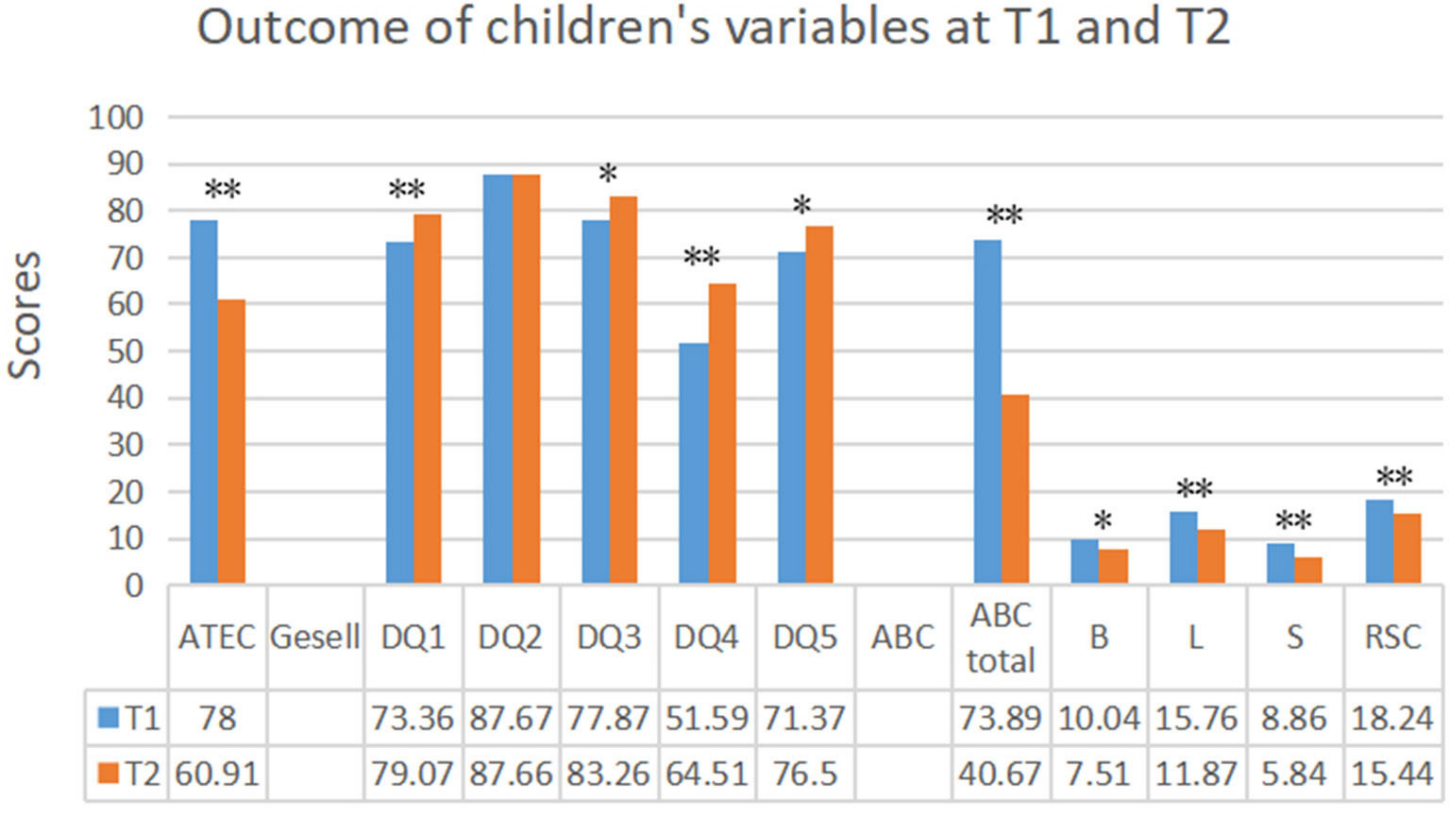
Outcome of children's variables at baseline (T1) and postintervention (T2). T1, preintervention; T2, postintervention; DQ1, Adaptive behavior; DQ2, Gross Motor behavior; DQ3, Language behavior; DQ4, Fine motor behavior; DQ5, Personal-social behavior; ATEC, The Autism Treatment Evaluation Checklist; ABC, The Autism Behavior Checklist; B, L, S, and RSC, the subscales of ABC. ^*^p < 0.05; ^**^p < 0.01.

The child outcome variables at baseline (T1) and postintervention (T2) are shown in Figure 2. Toddlers made statistically significant improvements in the Gesell subscales, including change in adaptive behavior DQ (*t* = 3.01, *p* = 0.004), language behavior DQ (*t* = 5.16, *p* < 0.001), fine motor behavior DQ (*t* = 2.23, *p* = 0.029) and personal-social behavior DQ (*t* = 2.61, *p* = 0.011), but they did not exhibit a significant change in Gesell gross motor behavior DQ before and after intervention (*t* = 0.01, *p* = 0.994). The subscale and total ABC scores (*z* = 6.20, *p* < 0.001) significantly decreased from intake to T2. In addition, the ATEC indicated that PCBI was effective, and the score decreased in this period of intervention (*t* = 9.05, df = 69, *p* < 0.001, Cohen's *d* = 0.74).

### Parent Outcomes

**Figure 3:**
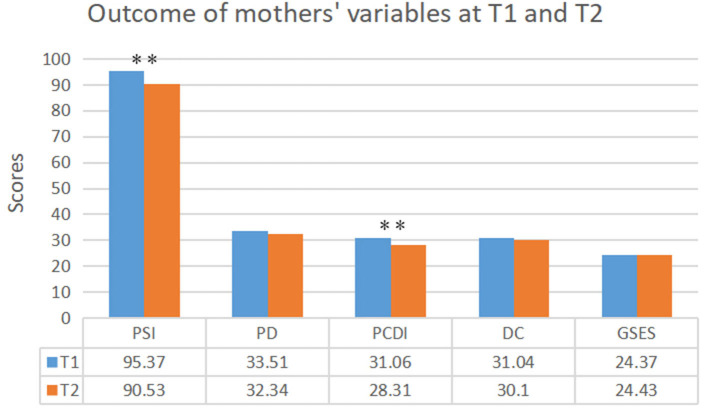
Outcome of mothers' variables at baseline (T1) and postintervention (T2). T1, preintervention; T2, postintervention; PSI-SF, The Parenting Stress Index Short Form; PD, PCDI, and DC, the subscales of PSI-SF; GSES, The General Self-Efficacy Scale. ^**^p < 0.01.

Figure 3 presents the means and standard deviations for mother outcomes at T1 and T2. Paired *t*-tests clearly revealed that during PCBI, parenting stress decreased in the total level of PSI (*z* = 2.86, *p* = 0.004) as well as on the mother-child dysfunctional interaction (*z* = 3.43, *p* = 0.001) subscale. However, no significant change was observed for the mother's self-efficacy level at preintervention and postintervention (*z* = 0.99, *p* = 0.318).

### Mother-Child Interaction Outcomes

Table 1 presents the change in mother-child interaction coding data for mothers and children. During PCBI, the rate of mothers' positive engagement (*z* = 2.59, *p* = 0.010) significantly increased, and mothers' disengagement (*z* = 3.24, *p* = 0.001) decreased. The rate of child positive engagement (*z* = 5.52, *p* < 0.001) also showed a statistically significant increase, while the degree of child-object engagement (*z* = 4.38, *p* < 0.001) was reduced. In addition, mother-child dyadic synchrony was significantly improved after intervention (*z* = 5.44, *p* < 0.001).

**Table 1:** Outcome of mother-child interaction variables at baseline (T1) and postintervention (T2).

	**T1**	**T2**	**Change scores**	***z***	***P***
**Mothers**					
Positive engagement	53.54 ± 8.78	56.66 ± 5.29	3.11 ± 8.41	2.59	0.010
Negative engagement	3.81 ± 6.80	1.96 ± 4.04	−1.86 ± 7.52	1.57	0.117
Disengagement	2.50 ± 3.94	1.17 ± 1.98	−1.33 ± 3.21	3.24	0.001
**Children**					
Positive engagement	9.16 ± 8.29	16.30 ± 11.47	7.14 ± 9.39	5.52	<0.001
Negative engagement	3.46 ± 8.97	3.06 ± 7.79	−0.40 ± 10.19	0.20	0.845
Disengagement	5.51 ± 8.04	5.94 ± 7.28	0.43 ± 8.93	0.09	0.932
Child-object engagement	41.81 ± 13.17	34.54 ± 12.79	−7.27 ± 14.82	4.38	<0.001
Dyadic engagement	9.03 ± 8.34	16.00 ± 11.56	6.97 ± 9.42	5.44	<0.001

*Data are expressed as the mean ± SD. T1, preintervention; T2, postintervention*.

### Summary of Hierarchical Regression Analysis for Variables Predicting the Intervention Effect (Change in ATEC Score)

As shown in Table 2, hierarchical multiple regression analyses of the children's and mothers' factors revealed that the completion level and change in the PSI score contributed significantly to the intervention effect (change in ATEC score). The completion level explained ~70% of the change in the ATEC score, with a change in the PSI score making a marginally unique contribution to the change in the ATEC score after controlling for the completion level.

**Table 2:** Summary of hierarchical regression analysis for children's and mothers' factors predicting intervention effect (change in ATEC score).

**Predictor variables (factors of mothers and children)**	**Model 1**			**Model 2**			**Model 3**		
	**B**	**SE B**	**β**	**B**	**SE B**	**β**	**B**	**SE B**	**β**
Mothers' age	0.33	0.50	0.08	0.18	0.26	0.05	0.19	0.25	0.05
ABC total score T1	0.07	0.04	0.21	−0.01	0.02	−0.03	−0.12	0.02	−0.05
Change in Children's DQ	−0.07	0.15	−0.06	0.04	0.08	0.03	0.05	0.08	0.04
Completion level				−15.69	1.27	−0.87^**^	−15.02	1.25	0.83^*^
Change in PSI score							0.17	0.07	0.17^**^
*R^2^*		0.06			0.74		0.76		
*F*		1.25			41.45^**^		37.41^**^		
*R^2^* change		0.06			0.68		0.03		
*F* change		1.25			152.60^**^		6.32^*^		

* *p < 0.05*;

** *p < 0.01*.

The regression on mother-child interaction variables indicated that change in child positive engagement and change in mother-child dyadic synchrony contributed significantly to the change in the ATEC score (Table 3). Change in child positive engagement explained ~30% of the change in the ATEC score, with change in mother-child dyadic synchrony making a significant contribution to the model.

**Table 3:** Summary of hierarchical regression analysis for mother-child interaction factors predicting intervention effect (change in ATEC score).

**Predictor variables (mother-child interaction factors)**	**Model 1**			**Model 2**			**Model 3**		
	**B**	**SE B**	**β**	**B**	**SE B**	**β**	**B**	**SE B**	**β**
Change in mother negative engagement	−0.04	0.26	−0.02	-1.16	1.26	−0.55	-0.79	1.15	−0.38
Change in mother disengagement	0.33	0.61	0.07	-0.83	1.36	−0.17	-0.70	1.24	−0.14
Change in child negative engagement	0.27	0.20	0.17	-0.01	0.18	−0.01	-0.15	0.17	−0.10
Change in child disengagement	0.04	0.23	0.02	-0.19	0.20	−0.11	-0.23	0.18	−0.13
Child-object engagement	0.18	0.14	0.17	-0.02	0.13	−0.02	0.26	0.13	−0.24
Change in mother positive engagement				-0.94	1.25	−0.50	-0.51	1.14	−0.27
Change in child positive engagement				-0.94	0.20	−0.56^**^	-0.45	0.22	−0.29^*^
Change in mother-child dyadic synchrony							-0.93	0.26	−0.54^**^
*R^2^*		0.05			0.31		0.43		
*F*		0.63			4.01^**^			5.85^**^	
*R^2^* change		0.05			0.26			0.12	
*F* change		0.63			11.90^**^			13.22^**^	

* *p < 0.05*;

** *p < 0.01*.

### Performance of the Regression Models

Table 4 shows the importance rankings of the top 5 variables for different algorithms for predicting the efficacy of PCBI. The best performance was found for the SVM model (*R*^2^ = 0.75, RMSE = 0.59), and the worst performance was found for the RR model (*R*^2^ = 0.53, RMSE = 0.70). Based on *R*^2^ and RMSE, the performance of different models can be ranked from strongest to weakest as follows: SVM > RF > LASSO > RR. Therefore, the SVM was selected as the best regression model for this study. As shown in Table 4, different machine-learning algorithms produced similar but different rank orderings because each method fit a different type of regression model. Mothers' factors, especially the completion level and mothers' stress (the subscale and total score of PSI) at baseline, seemed to be consistently important predictors. In addition to maternal variables, mother-child dyadic synchrony at baseline, which was ranked second place by the SVM model, was an important predictor in this study.

**Table 4:** Model fits for different algorithms and variable importance rankings.

	***R*^**2**^**	**RMSE**	**Variable rank**				
			**1**	**2**	**3**	**4**	**5**
LASSO	0.59	0.63	Completion level	Mother negative engagement	DC score (T1)	GSES score (T1)	Dyadic emotion engagement (T1)
SVM	0.75	0.59	Completion level	Dyadic emotion engagement (T1)	Change in dyadic emotion engagement	DC score (T1)	Child positive engagement (T1)
RR	0.53	0.70	Completion level	DC score (T1)	PD score (T1)	Dyadic emotion engagement (T1)	Change in adaptive behavior DQ
RF	0.61	0.57	Completion level	Change in adaptive behavior DQ	Satisfaction	Child positive engagement (T1)	PSI score (T1)

*LASSO, LASSO regression; SVM, support vector machine; RR, ridge regression; RF, random forest; PD and DC, the subscales of the Parenting Stress Index Short Form*.

## Discussion

Our first outcome verified a finding of our previous work, namely, that PCBI is effective for children with ASD aged 13–30 months (8). Analyses of the changes over 12 weeks of PCBI suggest that toddlers, as a group, improved in their developmental and autism symptoms with respect to gains in adaptive behavior, language behavior, fine motor behavior and personal-social behavior skills and decreases in the ATEC, ABC and subscale scores. In addition, we used video-recorded mother-child interactions to investigate the behaviors of children and mothers and found a significant gain in the level of child positive engagement and a reduction in child-object engagement. This finding is consistent with a study of parent delivery of the Early Start Denver Model intervention, which also conducted a 12-week intervention and found improvement of developmental skills (39). Previous studies have demonstrated that children with ASD have unstable structures of dyadic interactions and may have difficulty sustaining positive emotional states (18). However, the dyadic regulation ability of children with ASD in this study improved significantly. These results indicate that PCBI is an effective intervention method that can not only raise children's developmental levels but also enhance their emotional regulation and effective social interaction abilities. In addition, these children will be followed up and reassessed every year to check if the improvements were consolidated and generalized to other fields of their everyday life and now this work is ongoing.

Our second finding was that parenting stress decreased after 12 weeks of intervention. Previous reports demonstrated that mothers of children with ASD often experience much more stress than mothers of children with any other health problems, and parenting stress is related to child outcomes (11, 40, 41). In addition, mothers' self-efficacy was found to be an important factor affecting mothers' stress by adjusting the influence of children's behavior problems on mothers' anxiety and depression. The less satisfied mothers of children with ASD are, the higher the levels of pressure they experience (13, 42, 43). Consistent with these studies, we also found a negative correlation between mothers' self-efficacy and stress, although no significant difference in satisfaction was found between baseline and postintervention. In addition, the mother-child interaction videotapes showed an improvement in mothers' interaction skills, such as an increase in the level of positive engagement and a reduction in the level of negative engagement. This result indicates that PCBI can not only contribute to reducing mothers' stress but also improve mothers' skills in interacting with their children.

The main purpose of this study was to investigate and obtain preliminary data on the characteristics of toddlers who are more likely to benefit from PCBI. Available evidence indicates that many factors may impact the outcome of the intervention, including the characteristics of the mother and child (44–48). Thus, this study analyzed the main predictors from the perspective of mothers and children. Both the machine learning algorithms and hierarchical multiple regression indicated that maternal factors, such as mothers' stress and completion level of training at home, might be associated with the outcome of the intervention. Interestingly, mothers' stress, which was a predictor in this study, often emerges as a predictor in other reports focused on different intervention models (12, 13, 49), suggesting that more attention should be paid to the stress of mothers of children with ASD. In addition, the regression analysis of this study showed that the change in PSI score mediated the completion level of training at home and the ATEC scores. This might be interpreted as indicating that mothers with less stress were better able to complete their homework; in other words, stress may have affected their ability to perform the training at home.

The results of machine learning algorithms and hierarchical multiple regression suggest that PCBI might be particularly beneficial to children with ASD who show good performance in mother-child dyadic synchrony. Thus, the efficacy of PCBI can be improved through the improvement of mother-child dyadic synchrony. Dyadic synchrony refers to the emotional and behavioral responsivity of the mother and the child to each other and can make a positive contribution to children's development (50). The potential mechanism of this result might be that the improvement of mother-child dyadic synchrony may improve children's development as well as their behavior and communication ability. In addition, according to previous studies that demonstrated that the maternal psychological state, such as depression and stress, contributes to poorer dyadic synchrony (50), two crucial predictors in this study, mother-child dyadic synchrony and mothers' stress, may have mutual influences on each other and work together to affect the efficacy of PCBI. Consistent with our findings, Hobson et al. (51) indicated that parent-child dyadic synchrony was a mediating factor to predict child communication outcomes. Guo et al. (18) highlighted that children with ASD were more likely to exhibit emotion dysregulation; the problem of emotion dysregulation should be considered when an intervention is implemented. Our results indicated that mother-child dyadic synchrony was a crucial factor in predicting and monitoring the efficacy of the intervention that should be considered in future clinical interventions.

This study has a number of strengths, including the focus on both maternal and child characteristics as well as the analysis of video-recorded parent-child interactions and the use of machine learning algorithms. However, this study also has several limitations. First, the caregivers investigated in this study were the mothers of the children with ASD; the results cannot be generalized to fathers. Second, PCBI is the main intervention for children of this age in our team, and all the participants received PCBI without a control group; thus, it is unclear whether the findings are specific to responses to PCBI. It would be necessary to conduct a randomized control trial to test whether these predictors response to the PCBI only. Third, this study focused on short-term efficacy and factors likely to affect children's response to intervention. Longitudinal studies are needed to determine whether gains are maintained after a number of years and translate to meaningful cognitive, academic and social differences between children who accessed the intervention and those who did not. It is also necessary to analysis of each individual profile in our future work to provide a more specific intervention to each child.

In conclusion, this study indicated that mother-child dyadic synchrony, parenting stress of mothers and the completion level of training at home are important predictors of PCBI efficacy. In addition, the findings suggested that PCBI can contribute to reducing mothers' stress and improve the interaction skills between mothers and children with ASD.

## Data Availability Statement

The raw data supporting the conclusions of this article will be made available by the authors, without undue reservation.

## Ethics Statement

The studies involving human participants were reviewed and approved by the Nanjing Brain Hospital affiliated with the Nanjing Medical University Ethics Committee, and informed consent was obtained from the parents or legal guardians of the participants. Written informed consent to participate in this study was provided by the participants' legal guardian/next of kin.

## Author Contributions

JW and XK conceived and designed the study. LF, JW, MF, XX, TX, JF, NQ, CL, and YD performed the experiments. LF and JW analyzed experimental results. LF wrote the paper. LF, JW, and XK reviewed and edited the manuscript. All authors read and approved the manuscript.

## Conflict of Interest

The authors declare that the research was conducted in the absence of any commercial or financial relationships that could be construed as a potential conflict of interest.
